# Impact of Surfactants on Silk Fibroin Self-Assembly at the Air–Water Interface

**DOI:** 10.3390/polym17040529

**Published:** 2025-02-18

**Authors:** O. Yu. Milyaeva, R. Miller, G. Loglio, A. R. Rafikova, Z. Wan, B. A. Noskov

**Affiliations:** 1 Department of Colloid Chemistry, St. Petersburg State University, Universitetsky pr. 26, 198504 St. Petersburg, Russia; nastya.rafikova.2000@mail.ru (A.R.R.); b.noskov@spbu.ru (B.A.N.); 2 Institute of Condensed Matter Physics, Technische Universität Darmstadt, D-64289 Darmstadt, Germany; reinhard.miller@pkm.tu-darmstadt.de; 3 Institute of Condensed Matter Chemistry and Technologies for Energy, 16149 Genoa, Italy; giuseppe.loglio@ge.icmate.cnr.it; 4 School of Food Science and Engineering, South China University of Technology, Guangzhou 510640, China; zhiliwan@scut.edu.cn

**Keywords:** silk fibroin, air–water interface, surfactant, adsorption layers, dilational surface visco-elasticity, surface ellipsometry

## Abstract

Silk fibroin (SF)-based materials attract significant interest because of their biocompability and great diversity of possible morphologies. One of the approaches to obtain SF materials is the use of an air–water or oil–water interface as a template for protein self-assembly. Surfactants can change the surface properties of adsorbed SF layers by promoting or preventing the formation of SF fiber networks. This study focuses on the influence of two typical ionic surfactants, cationic cetyltrimethylammonium bromide (CTAB) and anionic sodium dodecyl sulfate (SDS), on the dynamic properties of SF layers adsorbed at the air–water interface. The dynamic surface elasticity, surface tension, ellipsometric angle Δ, and the film thickness were measured as a function of the surface age and surfactant concentration. The morphology of the layers was evaluated by atomic force microscopy (AFM). For the adsorption layers of globular proteins, the main effect of the surfactants consists in the protein unfolding at high concentrations and in a decrease in the electrostatic adsorption barrier. In the case of SF layers, CTAB and SDS strongly influence the protein aggregation at the air–water interface. Regardless of the sign of the surfactant charge, its addition to SF solutions results in a decrease in the surface elasticity and the destruction of the ordered structure of protein fibers at concentrations higher than 1 × 10^−4^ M. With the further increase in the surfactant concentration, the thread-like aggregates disappear, the packing of thin fibers becomes less tight, a uniform layer disintegrates into separate islands, and finally, the protein is displaced from the interface.

## 1. Introduction

Silk fibroin (SF) extracted from silk worms (*Bombyx mori*) is one of the most common natural biomaterials [1,2,3,4,5,6]. Due to its high biocompatibility, controllable biodegradability, mechanical properties, and variety of possible structures, it is widely used in tissue engineering [5,7,8] and drug delivery [9,10,11]. The diversity of applications determines the diversity of SF-based materials. They can be divided into two groups of almost two-dimensional materials, like thin films and membranes, and three-dimensional ones such as fibers, microparticles, and hydrogels [6,12,13,14].

Most of these materials are highly ordered since SF easily undergoes self-assembly processes [1,6,14,15,16]. The formation of supramolecular structures is governed by the combination of hydrophobic forces and hydrogen bonding as a result of some specific repetitive motifs in the protein primary structure. The SF molecule consists of one heavy (391 kDa) and one light chain (25 kDa) linked together by disulfide bonds [4]. The heavy chain primarily contains glycine (45.9%), alanine (30.3%), and serine (12.1%) [17]. These amino acids are organized into repetitive hydrophobic motifs like GAGAGS or GAGAGY/GAGAGVGY [18]. The interactions between these regions give rise to β-sheet formation and can induce transitions between three possible crystalline structures: silk I, silk II, and silk III [19,20,21]. These processes can be promoted by the addition of surfactants via a combination of hydrophobic interactions between SF chains and hydrophobic groups of the surfactant and dehydration or the “salt-out” effect [1,22,23]. Therefore, the formation of SF-based materials and their morphological features can be controlled by the addition of surfactants.

In particular, the addition of surfactants allows for a significant acceleration of the SF hydrogel formation and the improvement of its mechanical properties [1,5,22,23,24,25,26,27,28,29,30,31,32]. SF gelation can be considered as a nucleation and growth process starting from the formation of small nuclei with the subsequent growth of β-crystallites, followed by the development and branching of nanofibers, leading finally to single domains or domain networks [1,32]. SANS studies suggest that the surfactant-induced hydrogels contain β-sheet domains distributed in the amorphous random-coil matrix [33]. The assembly of β-sheet crystallites and random-coil matrices can lead to the formation of a denser network in the presence of a surfactant [1,22,23]. Almost all types of surfactants reduce the gelation time. This effect can be more pronounced for ionic surfactants as compared to non-ionic and zwitterionic ones [22,23,24,25,27]. In some cases, strong interactions of the negatively charged SF with cationic surfactants may lead to aggregation instead of gelation [22,29]. The use of biosurfactants such as sophorolipids and phospholipids is usually considered as an alternative to the application of conventional synthetic surfactants due to the better biocompatibility in the former case [26,28,30,31,33,34,35]. Note that in addition to the formation of hydrogels, the usage of surfactants can also facilitate the extraction of SF from natural raw material [36,37,38,39], ensure the production of micro- and nanoparticles with given properties [40,41], or regulate SF interactions with other components of composite materials [42,43,44].

SF itself is also characterized by a noticeable surface activity [45,46,47,48,49,50,51,52,53,54]. Due to its amphiphilic nature, SF is able to form micelle-like aggregates [54] and decrease the surface tension by strongly adsorbing at various interfaces [47,48,49,50,51,52,53]. The behavior of SF in the surface layer is influenced by a large set of parameters such as concentration, pH, and ionic strength. Even at small concentrations of about 0.002 mg/mL, SF considerably enhances the surface coverage and can form continuous two-dimensional networks of fibers at the interface [50,51]. At concentrations higher than 0.01 mg/mL, one can also observe, besides networks, thicker ribbons forming a structure like a branched tree [55]. At even higher concentrations, a layer of numerous interconnected fibers with a thickness of about 40 nm is formed. All surface structures mentioned above lead to a high dynamic surface elasticity and are able to stabilize foams and emulsions [48,49,50,51,52,53]. The high values of the surface elasticity can be attributed to the rearrangement of silk I to helical silk III, which is specific to SF at the air–water interface [20,21] or to laminated silk II. Both modifications are characterized by a high amount of β-sheet crystallites [20,56].

Similarly, regarding the surfactant’s impact on SF hydrogels, one can expect that the surfactant molecules can also affect the SF assembly in the surface layer, promoting β-sheet formation. It was shown that interactions of globular proteins with surfactants below their critical micelle concentration (CMC) influence the protein surface activity and their tertiary structure [57,58]. When the surfactant concentration approaches the CMC, the protein can be completely displaced from the surface layer. The latter effect was observed by Jayawardane et al. [47] and Qiao et al. [50,59] for SF and SDS at solid–liquid, air–water, and oil–water interfaces. Below the CMC, it was shown that conventional ionic surfactants penetrate the preadsorbed SF layer [47]. For fluid interfaces, the incorporation of surfactants into the SF adsorption layer increases the toughness of the interfacial networks and improves the stability of SF-based dispersed systems [50,59].

The stabilization or destabilization of dispersed systems, such as emulsions and foams, depends on surface properties. In these systems, the barriers for coalescence correlates with the rheological properties of the surface layers [60,61,62]. The fabrication and functioning of most SF-based materials involve the use of large interface boundaries [17,63]. For example, surface properties play a crucial role in obtaining nanostructured thin coatings [17,64], as well as in producing nano- and microparticles through the SF’s self-assembly on the oil–water interface in emulsions or microemulsions [40,41,62,65]. The control of interfacial properties by the surfactant’s addition can be useful for the creation of new materials with specific cellular or tissue responses.

Even though the properties of SF adsorption layers strongly influence the behavior of foams and emulsions containing this protein and are important for the production of SF-based nanomaterials, information on mixed surfactant/SF adsorption layers is quite limited. This study aims to elucidate the impact of two conventional ionic surfactants (CTAB and SDS) on the dynamic surface properties of the SF solutions using mainly measurements of the dilatational surface rheology. This technique proved its effectiveness in studies of the surface properties of complex fluids [66,67]. In this work, the dynamic surface elasticity, surface pressure, film thickness, and the adsorbed amount were determined in the course of different formation steps of the mixed adsorption layer. The layer properties near equilibrium were characterized by surface compression isotherms and AFM images.

## 2. Materials and Methods

Silk fibroin was isolated from fresh domestic *Bombyx mori* cocoon shells supplied by a Russian farm cooperative. The standard procedure described in [68] was used to obtain protein stock solutions. Sericin was removed by boiling the cocoon shells in 0.2 wt% Na_2_CO_3_ for 30 min. Afterward, the degummed SF was rinsed with deionized water, dried for one day at room temperature, and dissolved in a 9.3 M LiBr solution at 60 °C for 4 h. The obtained solution was dialyzed against water using a cellulose membrane (Sigma-Aldrich, Germany) for three days. As the final step, the solution was centrifuged two times (16,000× *g*, 4 °C, 30 min) to remove undissolved traces. The solution concentration after centrifugation (1 wt%) was determined by a gravimetric analysis. The aqueous protein solutions were stored in a refrigerator for no more than 20 days at a temperature of 4 °C. The investigated solutions in phosphate buffer were prepared from a stock solution of silk fibroin by dilution. NaH_2_PO_4_ and Na_2_HPO_4_ (Sigma Aldrich, Taufkirchen, Germany) were used to prepare the buffer solution with an ionic strength of 0.02 M and pH of 7. All measurements were carried out at a constant protein concentration of 0.02 mg/mL. The surfactant concentration varied from 1 × 10^−6^ M to 1 × 10^−2^ M. SDS and C_16_TAB were purchased from Sigma-Aldrich, Germany. CTAB was recrystallized twice from a mixture of ethylacetate and ethanol. SDS was recrystallized twice from ethanol. Triply distilled water was used to prepare all the solutions.

The dynamic surface elasticity and dynamic surface tension were determined by the oscillation ring method described elsewhere [69]. Briefly, the method allows for measurements of the surface tension response to periodical changes in the surface area. The periodical up-and-down movement of a roughed glass ring, which was partially immersed into the liquid, along its axis induces surface area oscillations leading to subsequent changes in the surface tension. The surface tension was measured by the Wilhelmy plate method. If the surface deformations are sinusoidal, the modulus of the dilatational surface elasticity can be determined by the following relation:

(1) $$|\varepsilon |=\frac{\Delta \gamma }{\Delta A/A}$$

where │ε│ is the modulus of the dilatational surface elasticity, ∆γ is the amplitude of the surface tension oscillations, and ∆A/A is the relative amplitude of the surface area changes.

The complex dynamic surface elasticity can be represented as the sum of the real and imaginary parts. For SF adsorption layers, the imaginary part of the dynamic dilatational surface elasticity was much less than the real one, so the data represented below are only the modulus values of the dynamic surface elasticity. All measurements were performed at a constant oscillation frequency of 0.05 Hz and a relative surface area change of 5%.

An ISR instrument equipped with a Wilhelmy plate (KSV NIMA, KSV Instrument Ltd., Helsinki, Finland) was employed to measure the compression isotherms of SF adsorption layers. The surface tension was measured with an accuracy of ±0.2 mN/m. After adding the SF solution to the Langmuir trough, the adsorption layer was compressed at a compression rate of 10 mm/min.

The surface coverage was characterized by ellipsometric data. Kinetic dependences of the ellipsometric angles Δ and ψ were obtained using the Multiskop null ellipsometer (Optrel GBR, Berlin, Germany) at a single wavelength of 632.8 nm and a fixed angle of incidence of 49° (close to the Brewster angle). When elliptically polarized light is reflected from the liquid surface, the polarization changes depending on the optical characteristics of the interface. These changes are described by two ellipsometric angles, ψ and Δ, which are related to complex reflection coefficients r_p_ and r_s_ of the parallel and perpendicular light components as follows:

(2) $$\frac{r_{p}}{r_{s}}=\tan\psi e^{i\Delta }$$

The ratio of the reflection coefficients depends on the wavelength of the incident light, the angle of incidence, the optical properties of the coexisting bulk phases, and the thickness and optical properties of the surface layer. For a special model of a thin homogeneous layer between two bulk phases, the refractive index and thickness of the layer can be calculated from the ellipsometric angles [70,71].

The surface concentration Γ of the single solute is approximately proportional to Δ_surf_ = Δ − Δ_0_, where Δ_0_ is the ellipsometric angle for pure water. The de Feijter equation can be applied to estimate the adsorbed amount of the protein [71]:

(3) $$\Gamma =\frac{\tau (n^{s}-n_{H_{2}O})}{dn/dc}$$

where τ is the layer thickness, n^s^ is the refractive index of the surface layer, n_H2O_ is the refractive index of water, and *dn*/*dc* is the refractive index increment of the protein solution.

The micromorphology of the SF and SF/surfactant mixed surface layers was studied using an atomic force microscope (NT-MDT, Moscow, Russia) in a semi-contact mode. The layers were transferred onto a mica surface from the air–water interface by the Langmuir–Schaeffer method after 12 h of liquid surface formation. After that, the samples were dried in a desiccator for several days at room temperature.

## 3. Results

### 3.1. Dynamic Surface Elasticity and Dynamic Surface Tension

**Figure 1:**
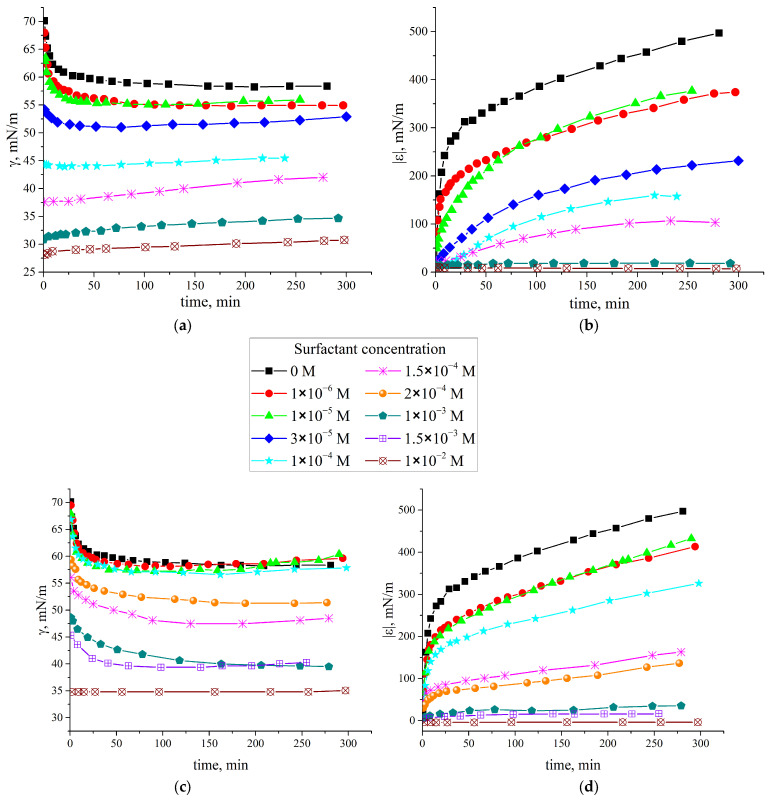
Kinetic dependencies of the dynamic surface tension (**a**,**c**) and the modulus of the dynamic surface elasticity (**b**,**d**) of 0.02 mg/mL SF solutions with addition of CTAB (**a**,**b**) and SDS (**c**,**d**), respectively, at surfactant concentrations of 0 M (black squares), 1 × 10^−6^ M (red circles), 1 × 10^−5^ M (green triangles), 1 × 10^−4^ M (blue diamonds), 1.5 × 10^−4^ M (cyan stars), 2 × 10^−4^ M (pink snowflakes), 1 × 10^−3^ M (orange circles), 1.5 × 10^−3^ M (violet crossed circles), and 1 × 10^−2^ M (brown crossed diamonds).

The dynamic surface tension and dynamic surface elasticity of fibroin/surfactant solutions were measured as a function of the surface age and surfactant concentration (Figure 1) and at a fixed protein concentration of 0.02 mg/mL. At this concentration, the pure fibroin tends to form two types of coexisting structures in the surface layer [51]. The first type is a well-developed and almost two-dimensional network of numerous branched thin fibers with a mesh size of several hundred nanometers. The second one consists of larger ribbons resembling a branched tree.

Such morphology corresponds to the highest values of surface elasticity for pure fibroin solutions in the concentration range from 0.0005 to 0.2 mg/mL. Its values approach 500 mN/m (Figure 1b,d), which is about eight times higher than typical values for globular protein solutions. This feature can be attributed to the SF rearrangement from Silk I to helical Silk III or laminated Silk II with an increase in the amount of β-sheet crystallites [21,56]. The surfactant’s addition can influence this process and change the dynamic surface properties. The influence of surfactant additions is noticeable already at concentrations of about 1 μM, at which the surface tension of pure surfactant solutions is close to that of pure water [69]. The dynamic surface tension and dynamic surface elasticity decrease monotonically under the influence of the surfactants. This effect is observed for both SF solutions with added CTAB (Figure 1a) and SDS (Figure 1c), respectively. At a relatively low surfactant content, the dynamic surface elasticity decreases with the concentration (Figure 1b,d) whereas the kinetic dependences of the surface tension remain almost unchanged. The dynamic surface elasticity close to equilibrium drops from 500 mN/m to 400 mN/m and to 350 mN/m for 1 × 10^−6^ M solutions containing CTAB and SDS, respectively. The rate of change of the surface properties increases with an increase in the surfactant concentration. At CTAB concentrations of 3.5 × 10^−5^ M and SDS concentrations of 2 × 10^−4^ M, a relatively high surface pressure is established already a few minutes after the surface formation. The dynamic surface elasticity decreases from 500 mN/m for pure SF solutions to almost zero at surfactant concentrations above the CMC. The surface tension also approaches values typical for pure surfactant solutions at corresponding concentrations.

**Figure 2:**
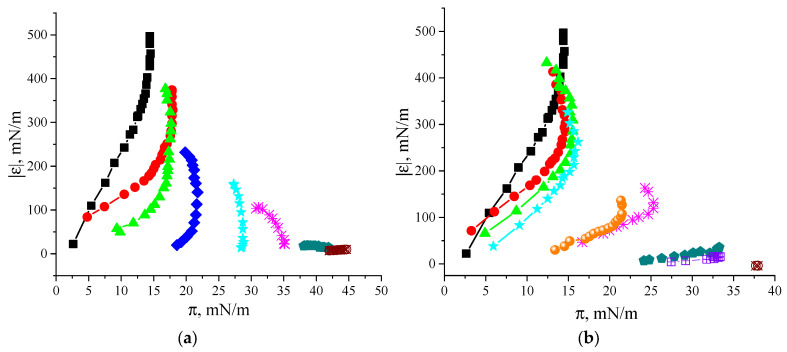
Modulus of the dynamic surface elasticity as a function of the surface pressure of SF solutions with addition of CTAB (**a**) and SDS (**b**), respectively, at surfactant concentrations of 0 M (black squares), 1 × 10^−6^ M (red circles), 1 × 10^−5^ M (green triangles), 1 × 10^−4^ M (blue diamonds), 1.5 × 10^−4^ M (cyan stars), 2 × 10^−4^ M (pink snowflakes), 1 × 10^−3^ M (orange circles), 1.5 × 10^−3^ M (violet crossed circles), and 1 × 10^−2^ M (brown crossed diamonds).

The dynamic surface properties change more gradually with the CTAB concentration (Figure 1a,b) than with the SDS concentration (Figure 1c,d). This feature becomes more evident while plotting the dynamic surface elasticity as a function of the surface pressure (Figure 2). In both cases, these dependences are shifted to higher values of the dynamic surface pressure with increasing surfactant concentration. This shift can be attributed to changes in the surface layer structure due to the surfactant’s impact and with a gradual SF displacement from the surface layer. At the same time, if SDS is added to the SF solutions, the obtained dependences below the surfactant CMC can be divided into three groups corresponding to concentrations from 1 × 10^−6^ M to 1 × 10^−4^ M, from 1.5 × 10^−4^ M to 2 × 10^−4^ M, and from 1 × 10^−3^ M to 1.5 × 10^−3^ M. It is possible to assume that these three groups correspond to three different types of adsorption layer structures.

### 3.2. Compression Isotherms

**Figure 3:**
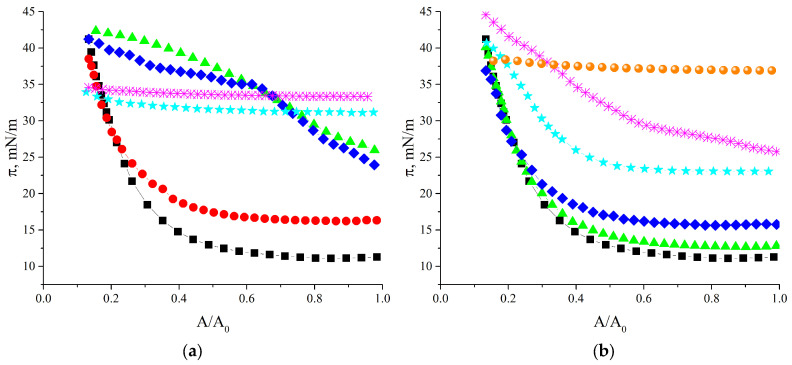
Compression isotherm of SF layers formed from SF solutions with the addition of CTAB (**a**) and SDS (**b**), respectively. Concentrations of the surfactants are as follows: 0 M (black squares), 1 × 10^−6^ M (red circles), 5 × 10^−5^ M (green triangles), 1 × 10^−4^ M (blue diamonds), 5 × 10^−4^ M (cyan stars), 1 × 10^−3^ M (pink snowflakes), and 1 × 10^−2^ M (orange circles).

The shape of the compression isotherms of SF layers is also affected by the added surfactant (Figure 3). Note that the initial points of the isotherms shift to higher surface pressures with the increase in surfactant concentration as a result of the corresponding changes in the steady state surface tension (Figure 1). Below the CMC, the compression isotherms can be divided into two characteristic regions with different slopes of the isotherm.

For SDS and CTAB concentrations less than approximately 1 × 10^−5^ M, the surface pressure is almost constant when the surface area decreases by less than 40%. It is known that small surfactant concentrations improve the mechanical properties of SF hydrogels [1,5,22,23,24,25,26,27,28,29,30,31,32]. A possible explanation is that interactions between surfactant molecules and protein chains facilitate the formation of small β-sheet domains, leading to the tighter packing of the fibers [1,24,32]. Such a structure results in a more homogeneous stress distribution and may prevent fast crack propagation [72]. A similar behavior can be characteristic for SF layers. If the layer consists of numerous cross-linked fibers, its compression leads to the formation of some close packed regions. The total SF network at the interface becomes denser but the surface pressure almost does not increase until the separate dense regions start to interact. The interaction of these regions with further compression can lead to a surface pressure increase.

If the surface area decreases to half of its original value, the surface pressure starts to increase more rapidly up to about 43 mN/m. It can be assumed that the start of compression leads only to some local changes in the layer thickness and its structure, resulting only in small changes in the surface pressure. The separate aggregates start to interact with further compression, leading to a faster increase in the surface pressure. At an SDS concentration of 5 × 10^−5^ M and CTAB concentration of 1 × 10^−4^ M, the surface pressure starts to increase at the very beginning of the compression, and beyond 35 mN/m, its changes slow down again. It can be assumed that at these concentrations, the layer consists of separate aggregates, which become closely packed at the beginning of the compression and start to interact with further compression. At higher surfactant concentrations, the surface pressure is almost insensitive to surface area changes due to the almost total displacement of SF by CTAB and SDS molecules from the interface.

### 3.3. Ellipsometry

**Figure 4:**
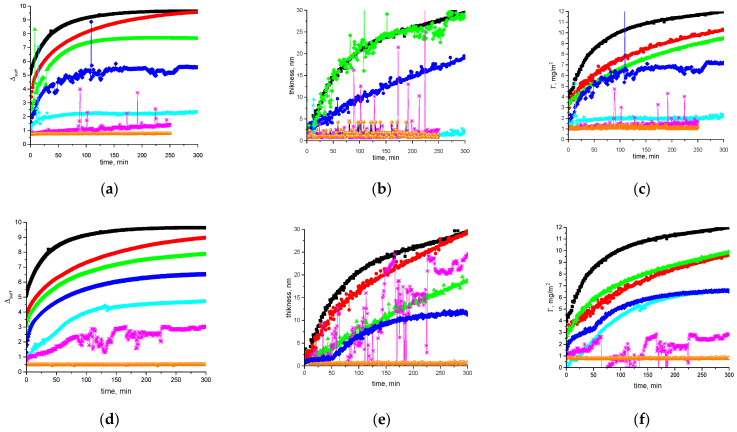
Kinetic dependencies of the ellipsometric angle Δ_surf_^0^ (**a**,**d**), film thickness (**b**,**e**), and adsorption (**c**,**f**) for 0.02 mg/mL SF solutions with addition of CTAB (**a**–**c**) and SDS (**d**–**f**), respectively. Concentrations of the surfactants are as follows: 0 M (black squares), 1 × 10^−6^ M (red circles), 1 × 10^−4^ M (green triangles), 1.5 × 10^−4^ M (blue diamonds), 1 × 10^−3^ M (cyan stars), 1.5 × 10^−3^ M (pink snowflakes), and 1 × 10^−2^ M (orange circles).

The influence of SDS and CTAB on the kinetic dependences of ellipsometric angles is similar (Figure 4). The ellipsometric angle Δ, which can be proportional to the adsorption value and the layer thickness, increases with the increase in surface age if the surfactant concentration does not exceed the CMC (Figure 4a,d). At the same time, the increase in surfactant concentration leads to a decrease in Δ. Near the CMC, the ellipsometric signal for mixed SF/surfactant solutions becomes indistinguishable from the results for pure surfactant solutions. The thickness of the layer decreases from about 28 nm to less than 2 nm (Figure 4b,e), and the adsorbed amount drops from 10 to 1 mg/m^2^ (Figure 4c,f). Another effect of the surfactant addition is the numerous fluctuations of the ellipsometric signal in the intermediate concentration range. It can be assumed that SF is not displaced from the surface layer to a significant extent in this range, but the continuity of the initial adsorption layer is destroyed. The pure fibroin layers are also not uniform enough, but the surface is covered by a network of thin SF fibers and the strong fluctuations of the ellipsometric signal are possible only at sufficiently lower bulk concentrations of the protein. After the surfactant’s addition at concentrations of 1 × 10^−4^ M (CTAB) and 1 × 10^−3^ M (SDS), one can see strong fluctuations of the ellipsometric signal with time, if the surface lifetime exceeds a certain critical value. A slight growth of the surfactant concentration results in fluctuations during the total observation period. Such behavior can be explained by the formation of separate aggregates at the interface, which are able to move freely along the interface [73,74]. The fluctuations are observed in a slightly broader concentration range of CTAB than of SDS.

### 3.4. Atomic Force Microscopy

**Figure 5:**
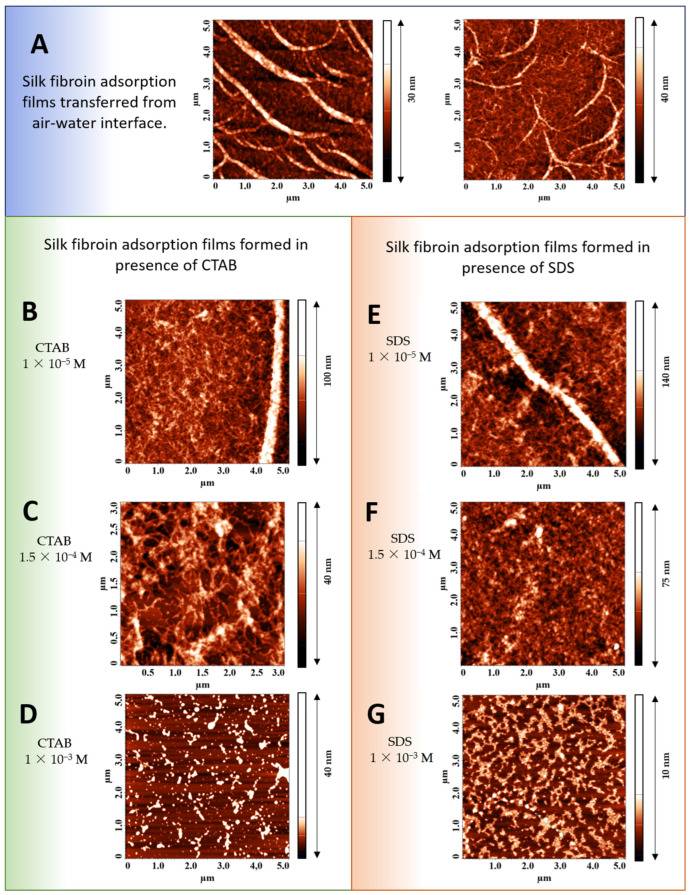
AFM images of surface layers formed in pure SF solutions (**A**) and SF solutions with addition of CTAB (**B**–**D**) and SDS (**E**–**G**) and transferred from the air–water interface onto a mica surface. Concentrations of the surfactants are as follows: 0 M (**A**), 1 × 10^−5^ M (**B**,**E**), 1.5 × 10^−4^ M (**C**,**F**), 1 × 10^−3^ M (**D**,**F**).

The AFM data corroborate the main assumptions above (Figure 5). SF adsorption layers at a concentration under study are characterized by the coexistence of branched thin fibers and thicker branched ribbons (Figure 5A). The size in the Z direction of these two structures corresponds approximately to 5 nm and 40 nm, respectively. The addition of small amounts of CTAB or SDS changes the layer structure. At concentrations close to 1 × 10^−5^ M, the number of ribbons decreases and they become less branched (Figure 5B,E). The subsequent increase in the surfactant concentration leads to changes in the layer morphology and the ribbons disappear (Figure 5C,F). The thin fibers become less uniform and can resemble a sponge. The mesh size increases from about 50 nm to 300–500 nm. The two-dimensional aggregates in the layer are weakly connected to each other via thin fibers. At the surfactant concentration of about 1 × 10^−3^ M, the fibers almost disappear and only some amorphous aggregates can be found in the layers at high CTAB and SDS concentrations (Figure 5D,G).

## 4. Discussion

The surfactant’s influence on the surface properties of fibrous protein solutions is less studied as compared with mixed solutions of globular proteins and surfactants [57,58,66]. In the latter case, the impact of cationic and anionic surfactants on the surface properties of protein solutions is rather different due to distinctions in the electrostatic interactions. The addition of an oppositely charged surfactant to globular protein solutions leads to the acceleration of changes in surface properties and the appearance of a local maximum of the surface elasticity. The nonmonotonic kinetic dependences of the surface elasticity are caused by the protein denaturation and partial displacement of the unfolded protein chains from the proximal region of the surface layer into the distal one in the form of loops and tails. The addition of a similarly charged surfactant can lead to a decrease in the rate of change of the surface properties and stabilization of the protein globular structure due to the surfactant’s penetration into the hydrophobic cavities of the globules. As a result, all the dependences of the surface elasticity become monotonic. At concentrations above the CMC, the protein is displaced from the surface by the surfactant regardless of the component charge.

Unlike the case of globular protein solutions, an acceleration of changes in surface properties is observed for mixed SF solutions with both CTAB and SDS. The influence of the electrostatic adsorption barrier becomes negligible due to relatively high SF concentrations. The adsorption of SF at a concentration of 0.02 mg/mL is almost insensitive to the increase in the solution’s ionic strength, corroborating the absence of a significant adsorption barrier [51]. Another distinction between the two systems is connected with the difference in structures of SF and globular proteins and the SF’s ability to form large aggregates. Fibrous SF consists of a hydrophobic heavy chain connected to a light hydrophilic chain by disulfide bonds [4]. The repetitive structures like GAGAGS, GAGAGY, and GAGAGV in the heavy chain allow for the formation of anti-parallel β-sheets due to hydrogen bonds; however, the surfactants can modify this process. In SF hydrogels, the degree of aggregation depends on the surfactant type [1]. For non-ionic and anionic surfactants, the increased dehydration results in an easier formation of β-sheet structures, while for cationic surfactants, the strong interactions between oppositely charged components sometimes lead to the formation of separate aggregates. At high anionic surfactant concentrations above the CMC, both monomers and micelles can interact with the SF chains, and the gelation time slows down due to the large electrostatic repulsion [1]. The influence of surfactants on SF layers at the air–water interface, similarly to the corresponding effects in the bulk phase, strongly depends on the concentration. Although the SF concentration in the bulk phase is insufficient for gelation, its concentration in the adsorption layer is high enough, and one can expect a similarity in the behavior of SF hydrogels and its adsorption layers.

**Figure 6:**
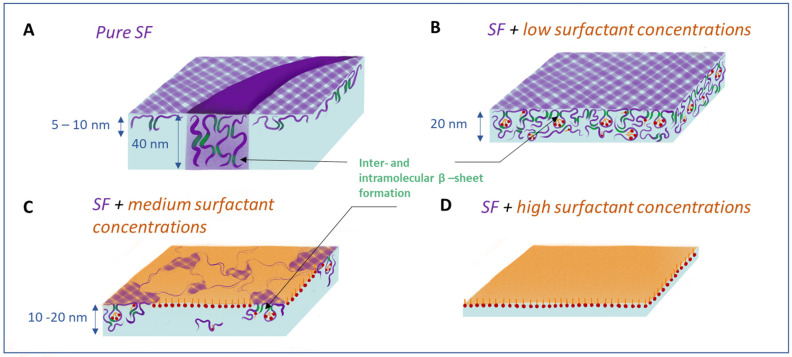
Scheme of SF surface layer structure transitions under the action of the surfactant.

When a large multi-block amphiphilic macromolecule like SF is adsorbed at the air–water interface, it can continue to rearrange in such a way that both hydrophobic and hydrophilic chains finally reach the most favorable environment. This process can result in an increase in the dynamic surface elasticity and surface pressure, leading to transitions from Silk I to helical Silk III or laminated Silk II with a high amount of β-sheet crystallites [20,56]. The variety of supramolecular structures such as threads, ribbons, and more complex surface aggregates (Figure 6A), which were observed by AFM (Figure 5A), respond differently to the surface deformation [51]. The pure SF adsorption layer is characterized by elasticities five times higher than those of globular protein solutions, which is comparable with the values for the layers of solid nanoparticles [75]. One can assume that the observed thick ribbons and the network of threadlike aggregates contain regions of laminated Silk II or helical Silk III with a high strength. A significant part of SF molecules in the ribbons probably corresponds to Silk III modification. This crystal structure involves an approximately hexagonal packing of protein molecules with a left-handed threefold helical chain conformation. Silk III modifications specifically arise at the air–water interface [20,21].

At surface pressures of about 16.7 mN/m, Silk III has an uniaxially oriented crystalline texture, with the helical axis oriented perpendicular to the plane of the layer [20]. It can be assumed that the large thickness of 40 nm of Silk III regions is a result of this orientation of the ribbons (Figure 6A). The structure of this kind coexists with threadlike aggregates, which are characterized by a relatively low thickness of about 5–10 nm.

The coexistence of these two types of structures of pure SF layers results in an average ellipsometric thickness of about 30 nm. At small surfactant concentrations, the mixed layer becomes more uniform as compared to the pure SF layers, and only a network of threadlike aggregates can be observed (Figure 6B). Similar to pure protein layers, one can expect an abrupt decrease in the layer thickness corresponding to a transition to networks of threadlike aggregates; however, the ellipsometric results show only a rather moderate decrease in the film thickness to approximately 20 nm. Therefore, one can assume that the increase in nucleation and formation of β-sheet structures under the influence of surfactants results in a layer growth mainly in the Z-direction (Figure 6B). When the ribbons disappear, the ratio between the Silk II and Silk III crystalline structure is shifted in the direction to Silk II, which is less rigid, and the dynamic surface elasticity decreases.

At even higher surfactant concentrations, especially CTAB, the continuous network is destroyed and replaced by some aggregates and free surfactant molecules (Figure 6C,D). The aggregates are connected between themselves via thin fibers. With a further increase in the surfactant concentration, the fibers disappear and one can observe only small separate amorphous particles. The destruction of the uniform layer can be a consequence of strong interactions between the two components when the formation of compact aggregates is more favorable as compared to the growth of the network. Previously, it was shown for hydrogels containing SF that in the case of oppositely charged components, the combination of electrostatic and hydrophobic interactions leads to the formation of compact SF particles and phase separations [22].

The increase in the surfactant concentration above the CMC causes an almost complete removal of SF from the surface layer, similarly to the observed effect for SF adsorbed at the liquid–solid interface [47]. Since the diffusion coefficient of small SDS and CTAB molecules is significantly higher than that of SF, they very quickly occupy the surface after its formation.

Information on the assembly of SF layers is expected to be of fundamental and practical interest for obtaining SF-based materials of a given morphology and thickness. The transferal of the SF layers from the air–water interface can be used for obtaining new ultrathin coatings of complex functionalized nanoarchitectures, leading to new opportunities in biomaterials engineering.

## 5. Conclusions

The dynamic surface properties of SF solutions are significantly influenced by the addition of CTAB and SDS. The increase in concentrations of both surfactants, regardless of their charge, leads to a decrease in the dynamic surface elasticity, dynamic surface tension, adsorbed amount, and film thickness as compared to SF layers in pure protein solutions. This decrease can be attributed to a gradual destruction of the ordered self-assembly structures in the surface layer, which can be confirmed by AFM. The surfactant increases the flexibility of SF chains and promotes intermolecular interactions between molecules and their self-assembly, finally leading to the formation of amorphous aggregates. In the first step, when the surfactant concentration is less than 1 × 10^−5^ M, the surfactant facilitates intra- and intermolecular β-sheet formation, resulting in a growth in the Z-direction of the initially almost two-dimensional network without the development of ribbons. Since these ribbons are observed in regions corresponding to a high Silk III content and lead to the high rigidity of the layer, their disappearance leads to a decrease in the dynamic surface elasticity. The surfactant adsorption and the gradual displacement of the protein results in a transition of a continuous layer to a layer of separate islands, which are initially interconnected via thin threads, become separated after that, and finally are displaced from the surface by the surfactant molecules.

## Data Availability

The data presented in this study are openly available in the article.
